# Dietary Supplementation of Vine Tea Ameliorates Glucose and Lipid Metabolic Disorder via Akt Signaling Pathway in Diabetic Rats

**DOI:** 10.3390/molecules24101866

**Published:** 2019-05-15

**Authors:** Jiamei Xiang, Qiuyue Lv, Fan Yi, Yanjun Song, Liang Le, Baoping Jiang, Lijia Xu, Peigen Xiao

**Affiliations:** 1 Institute of Medicinal Plant Development, Chinese Academy of Medical Sciences and Peking Union Medical College, Beijing 100193, China; xiangjiamei2014@163.com (J.X.); lqiuyue@126.com (Q.L.); eryue1024@163.com (Y.S.); llfxcy@163.com (L.L.); xiaopg@public.bta.net.cn (P.X.); 2 Key Laboratory of Bioactive Substances and Resources Utilization of Chinese Herbal Medicine, Ministry of Education, Beijing 100193, China; 3 Key Laboratory of Cosmetic, China National Light Industry, Beijing Technology and Business University, Beijing 100048, China; fantasyee@btbu.edu.cn

**Keywords:** vine tea, glucose and lipid metabolic disorders, Akt signaling pathway, metabolomics, type 2 diabetes

## Abstract

A traditional Chinese tea with many pharmacological effects, vine tea (VT) is considered a potential dietary supplement to improve type 2 diabetes (T2D). To investigate the effect and mechanism of VT on glucose and lipid metabolic disorders in T2D rats, Wistar rats fed a normal diet served as the normal control, while rats fed a high-fat diet combined with low-dose streptozotocin (STZ)-induced T2D were divided into three groups: The model group (MOD); the positive control group (MET, metformin at 200 mg/kg/d); and the VT-treated group (VT500, allowed to freely drink 500 mg/L VT). After four weeks of intervention, biochemical metrics indicated that VT significantly ameliorated hyperglycemia, hyperlipidemia and hyperinsulinemia in T2D rats. Metabolomics research indicated that VT regulated the levels of metabolites closely related to glucose and lipid metabolism and promoted glycogen synthesis. Furthermore, VT had a significant influence on the expression of key genes involved in the Akt signaling pathway, inhibited gluconeogenesis through the Akt/Foxo1/Pck2 signaling pathway, and reduced fatty acid synthesis via the SREBP1c/Fasn signaling pathways. In conclusion, VT has great potential as a dietary supplement to ameliorate glucose and lipid metabolic disorders via the Akt signaling pathway in T2D rats.

## 1. Introduction

In the past three decades, the incidence of diabetes mellitus has dramatically increased. Globally, there are approximately 415 million people with diabetes mellitus, which is considered a major cause of death [1,2]. Patients diagnosed with type 2 diabetes (T2D) account for more than 90% of all diabetic patients [1]. Regionally, Asia is a major contributor to the rapid emergence of T2D, with China and India as representatives [2]. The main features of T2D are hyperglycemia and insulin resistance in target organs, and as obesity and unreasonable dietary patterns become more prominent, hyperlipidemia often occurs in patients with T2D. Therefore, T2D is generally considered to be a chronic metabolic disorder. Reasonably, improving the metabolic dysfunction of glucose and lipid is an important direction for T2D prevention and treatment [3,4,5,6,7].

As a rapidly developing technology for expanding our understanding of biological processes, metabolomics is becoming an active research area in the biomedical field [8,9] and is widely used in mechanism research, disease diagnosis and the therapeutic evaluation of chronic metabolic diseases involving glucose and lipid metabolic disorders [10,11], such as obesity [12], diabetes [13], hyperlipidemia [14,15], and hypertension [16].

As a result of the rapidly increasing prevalence of T2D, intensive research on supplementary treatments for T2D has similarly increased [17]. Furthermore, natural products and functional foods are considered to have great potential for the supplementary treatment of T2D [18]. Tea is a representative of beverages with a long history of application and is believed to promote good health in traditional Asian medicine [19,20]. Many studies have focused on the potential of tea, especially teas with traditional pharmacological effects, as a dietary supplement therapy for T2D [21,22,23,24]. The safety of tea affords great advantages for a dietary supplement therapy; tea can be consumed for a long time with few negative side effects and little psychological stress. In addition, the market demand for tea as a supplement therapy for T2D is very large, and the development of related research will promote the development of the tea industry to a large extent, including the cultivation and management of tea. Vine tea (VT) is a type of functional tea widely used in China, derived from the dried leaves of *Ampelopsis grossedentata*, and it was listed as a new resource of food raw materials by the relevant Chinese government departments. (http://www.moh.gov.cn/) in 2014. Studies have revealed that VT has multiple pharmacological activities, such as anti-inflammatory [25], hepatoprotective [26], and antioxidant [27] effects. Previous studies from our laboratory indicated that VT and its main components significantly prevent insulin resistance in a cell model of insulin resistance [28,29] and have a significant preventive effect on insulin resistance and metabolic abnormalities in animal models fed a high-fat diet (HFD) [28,30]. However, whether VT can be used as a dietary supplement treatment for the hyperglycemia and hyperlipidemia of T2D requires more intensive research. Therefore, the ultimate aim of this experimental study was to clarify whether VT can be used as a dietary supplement for glucose and lipid metabolic disorders in a T2D rat model induced by a HFD combined with a small dose of streptozotocin (STZ) and to reveal the related mechanism of action of VT.

## 2. Results

### 2.1. Quality Control of VT

In VT, the content of dihydromyricetin (DHM), which is considered to be the major bioactive component in VT, can be higher than 20% [31,32]. Therefore, DHM was used as a reference standard to evaluate VT quality (Figure S1). Three samples were randomly selected for detection. The results showed that the DHM content of VT was 22% ± 3%.

### 2.2. Effect of VT on Food and Water Intake and Body, Liver and Epididymal White Adipose Tissue Weights in Rats with T2D 

In our research, a modeling method of a HFD combined with a small dose of STZ was used to induce a rat model of T2D. This method of modeling T2D directly influences the body weight (BW) and dietary intake of rats. Compared with the CON group, the food intake (FI) and BW of rats significantly decreased in the HFD group during the eight-week model establishment process, as shown in Figure S2A. Therefore, when the rats in the HFD group were randomly grouped into the model (MOD), metformin (MET), and VT (VT500) groups based on blood glucose level at the end of the eighth week of modeling experiment, the BW of rats in the CON group was greatly different from that of other groups. However, there was no significant difference in the BW of rats in the MOD, MET, and VT500 groups (Table 1 and Figure S2B).

After four-weeks of treatment with VT, compared with the MOD group, the BW gain and food utilization rate increased significantly in the VT500 group and the MET group, but the FI among the groups did not show significant differences (Table 1 and Figure S2C). The CON group consumed an average of 803.84 g of normal diet (ND) per rat. The MOD, MET, and VT500 groups consumed 825.4 g, 778.1 g, and 789.2 g of the HFD per rat, respectively. The results indicated that VT and MET can improve weight control in T2D rats (Table 1). The water intake data are shown in Figure S2D. The VT500 group ingested an average of 3526 mL of VT per rat, while the CON, MOD, MET groups ingested 1467 mL, 3700 mL, and 3238 mL of water per rat, respectively. The CON group was significantly different from the other groups in terms of water intake; water intake was slightly decreased in the VT500 and MET groups, but no significant difference was found compared with the MOD group.

In the present study, we found that VT also had a certain effect on the liver and epididymal white adipose tissue (WAT). The liver-to-BW ratio of the MOD group of rats was significantly increased compared with that of the CON group of rats, which may indicate edema, inflammation, or hypertrophy in the liver of the MOD group of rats. For all treatments, significant reductions in the liver-to-BW ratio were observed (Figure S2E). Moreover, significant increases in the epididymal WAT-to-BW ratio were discovered, which may be the cause of the increased weight gain in the MET group and VT500 group compared to the MOD group (Figure S2F).

### 2.3. Effect of VT on Serum Biochemical Indices

#### 2.3.1. VT Improves Dyslipidemia and Liver Function

**Figure 1:**
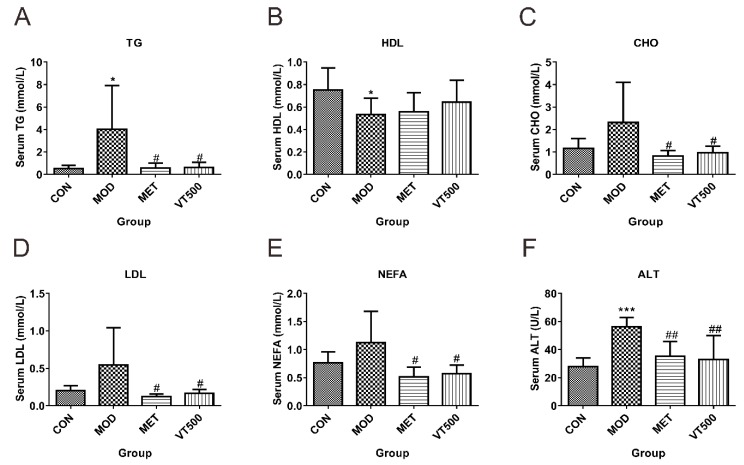
Effect of VT on biochemical indicators related to serum lipid and liver function in rats with T2D. Triglycerides (TG, **A**), high-density lipoprotein (HDL, **B**), cholesterol (CHO, **C**), low-density lipoprotein (LDL, **D**), nonesterified fatty acids (NEFA, **E**), and alanine transaminase (ALT, **F**) were measured at the end of four-weeks of treatment with VT. The data shown mean ± SD. * *p* < 0.05, *** *p* < 0.001 compared with the CON group; # *p* < 0.05, ## *p* < 0.01 compared with the MOD group, *n* = 6.

Most cases of T2D are accompanied by dyslipidemia, which is an important cause of T2D incidence and development and is also a treatable relevant aspect for subsequent cardiovascular complications that may develop in patients with T2D [33,34,35]. The liver is an important metabolic organ in the human body and directly affects the metabolism of glucose and lipids. The results showed that, in comparison to the CON group, the MOD group had appreciably increased levels of serum triglycerides (TG) (Figure 1A) and significantly decreased serum HDL levels (Figure 1B). Furthermore, the serum concentrations of cholesterol (CHO), low-density lipoprotein (LDL), and nonesterified fatty acids (NEFA) of the MOD group tended to be increased (Figure 1C–E). In contrast, the serum contents of TG, CHO, LDL, and NEFA were significantly reduced (Figure 1A,C,D,E) in the VT500 group, while the serum HDL level tended to be increased after VT treatment (Figure 1B). The results indicated that VT improved dyslipidemia in T2D rats. In addition, the serum ALT level of rats in the MOD group was significantly raised compared with the CON group, while VT treatment significantly decreased the ALT level, which suggested that VT has a protective effect on the liver (Figure 1F).

#### 2.3.2. VT Improves Glucose Metabolism Disorder and Insulin Resistance

**Figure 2:**
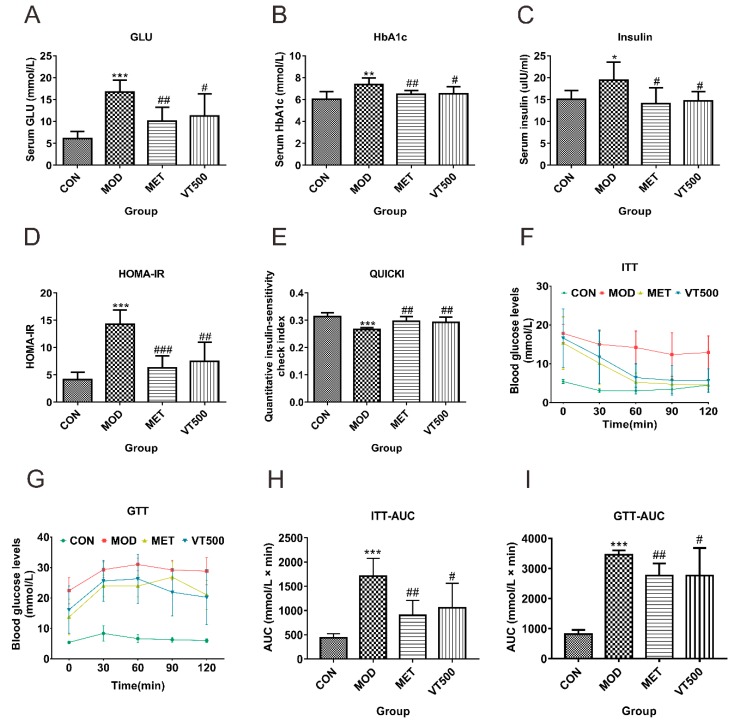
Effect of VT on serum glucose (GLU), glycated hemoglobin A1c (HbA1c), insulin, glucose tolerance and insulin sensitivity in rats with T2D. Fasting serum glucose (**A**), HbA1c (**B**), and insulin (**C**) were measured at the end of 4 weeks of treatment with VT, and the homeostasis model assessment of insulin resistance (HOMA-IR, **D**) index and the quantitative insulin sensitivity check index (QUICKI, **E**) were calculated. Blood was collected from the tail vein, and glucose levels were determined 0, 30, 60, 90, and 120 min after either insulin administration in the insulin tolerance test (ITT, **F**) or 50% glucose administration in the glucose tolerance test (GTT, **G**). The insulin area under the curve (AUC, **H**) and glucose AUC (**I**) was calculated. The data shown as the mean ± SD. * *p* < 0.05, ** *p* < 0.01, *** *p* < 0.001 compared with the CON group; # *p* < 0.05, ## *p* < 0.01, ### *p* < 0.001 compared with the MOD group, *n* = 6.

The level of glycated hemoglobin A1c (HbA1c) has been widely used as an important criterion for diagnosing diabetes and glycemic control in clinical practice because this metric is not affected by short-term fluctuations in blood sugar [36,37]. Therefore, we measured the levels of HbA1c, glucose (GLU), and insulin in the serum of rats from each group. Then, we calculated the homeostasis model assessment of insulin resistance (HOMA-IR) index and the quantitative insulin sensitivity check index (QUICKI). Our results showed that, compared to the CON group, the serum GLU, HbA1c, and insulin levels are the same as the HOMA-IR index in the MOD group were significantly increased (Figure 2A–D), while the QUICKI in the MOD group was significantly decreased (Figure 2E). To more objectively explore the influences of VT on impaired insulin sensitivity and glucose tolerance in T2D rats, the insulin tolerance test (ITT), and glucose tolerance test (GTT) were also performed (Figure 2F–I). We observed that the rats in the MOD group showed higher glucose tolerance and lower insulin sensitivity than that of the CON group. In conclusion, all of these findings demonstrated that the MOD group rats showed typical T2D symptoms of glucose metabolism disorder and insulin resistance.

Compared with the MOD group rats, the serum GLU, HbA1c and insulin levels were significantly decreased (Figure 2A–C) in VT-treated rats. The HOMA-IR index significantly decreased, and the QUICKI significantly increased after VT treatment (Figure 2D,E). Moreover, compared to the MOD group, VT-treated rats had a rapid decrease in blood glucose and were able to maintain a lower blood glucose level under insulin stimulation (Figure 2F,H). The GTT results also showed that the ability to maintain blood glucose homeostasis was enhanced by VT in T2D rats (Figure 2G,I). These findings suggest that VT has great potential as a supplementary therapy for T2D through enhancing insulin sensitivity and improving blood glucose regulation.

### 2.4. Metabolomic Analysis of the Role of VT on the Metabolic Disorder Induced by STZ Combined with HFD in Rats

**Figure 3:**
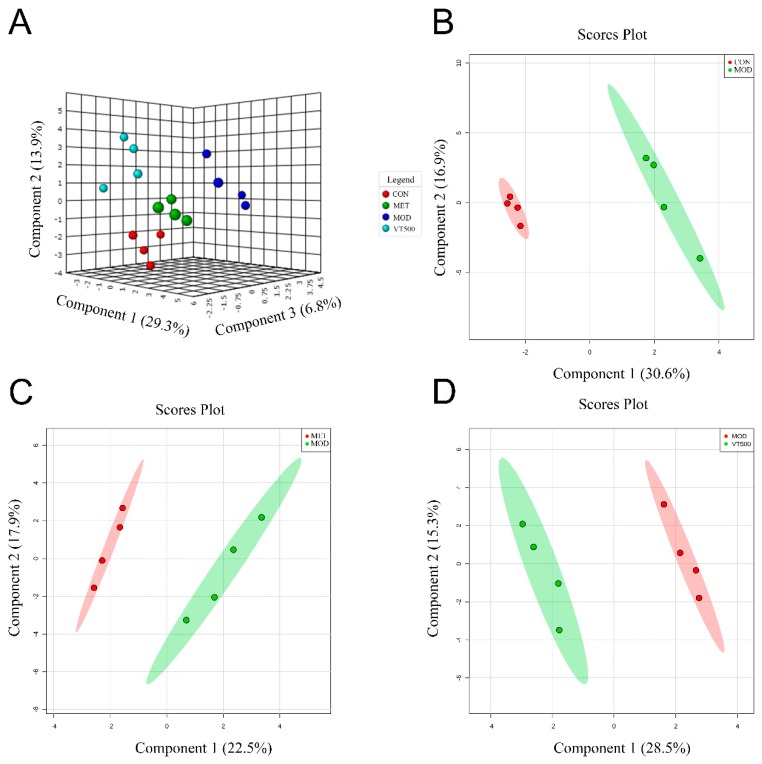
Effect of VT on liver metabolism in rats. Three-dimensional sparse partial least squares-discriminant analysis (sPLS-DA) of the CON, MOD, VT500 and MET groups (**A**); sPLS-DA score plots of all groups (**B**); sPLS-DA score plots of the MOD and MET groups (**C**); sPLS-DA score plots of the MOD and VT500 groups (**D**), *n* = 4.

LC-MS was used to qualitatively and quantitatively analyze the metabolites in the rat liver, and we identified a total of 174 metabolites and quantified them with standard chemicals. Sparse partial least squares-discriminant analysis (sPLS-DA) was used to overview the resemblance of metabolites among the four groups (Figure 3A). The result showed that the liver metabolites of the MOD rats were clearly separated from those of the CON rats (Figure 3B), indicating that metabolic disorder occurs in the MOD group. However, after the MET and VT interventions, the liver metabolites of rats were significantly changed, in contrast to the liver metabolites of the MOD group of rats (Figure 3C,D), suggesting that VT has a certain influence on liver metabolism.

**Figure 4:**
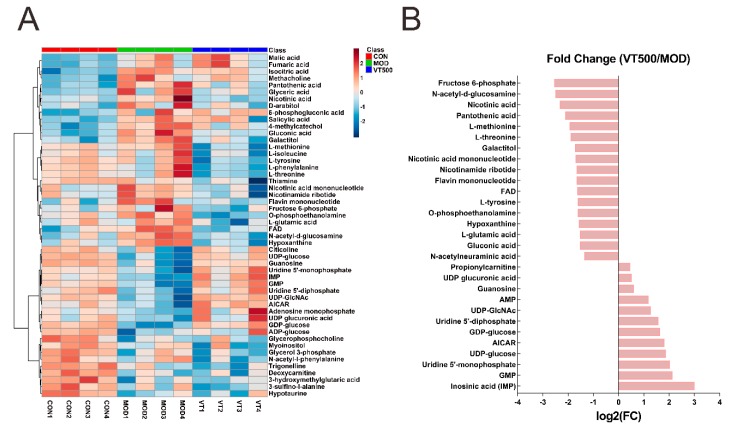
Effects of VT on the concentration of liver metabolites in rats. The heatmap of the 50 most impacted hepatic metabolites among the CON, MOD and VT500 groups (**A**); analysis of the changes in the content of 29 key hepatic metabolites (*p* < 0.05) between the VT500 group and MOD group (**B**).

To explore the effects of VT on liver metabolites, the 50 hepatic metabolites with largest changes in concentration among the CON, MOD and VT500 groups were selected to generate a heatmap (Figure 4A); the metabolites are shown as squares with carefully chosen color gradients to represent the normalized data. In comparison to the CON group, 43 key hepatic metabolites (*p* < 0.05) were prominent in all metabolites in the MOD group of rats, of which 22 metabolites showed a significant increase in levels, while the levels of the other 21 metabolites were significantly decreased. After VT treatment, 29 of the altered metabolites were considered key metabolites (*p* < 0.05). Among them, the absolute concentrations of 12 metabolites were increased; conversely, the levels of 17 metabolites were lower than that of the MOD group (Figure 4B).

### 2.5. VT Affects Purine, Amino Acid and Glucose Metabolism in the T2D Rat Liver

To further investigate the influence of VT in the metabolic pathway of T2D rats, KEGG metabolic pathway analysis was performed on the 29 key hepatic metabolites identified between the MOD and VT500 groups (Table S1). The results indicated that a total of 27 metabolic pathways were involved and suggested that VT intervention mainly affected the amino acid metabolic pathway, the purine metabolic pathway, and the glucose-related metabolic pathway, which are closely related to the glucose and lipid metabolism and hepatic insulin resistance in T2D rats [38,39].

Based on the results of the KEGG analysis, among the 29 key metabolites, we focused on several metabolites in the metabolism of amino acid, purine and glucose. We separately analyzed the concentration of several amino acids in the liver of the MOD rats and the VT500 rats and found a significant decrease in the levels of l-tyrosine, l-methionine, l-threonine, and l-glutamic acid after VT treatment. Moreover, l-phenylalanine and l-isoleucine exhibited a downward trend in the VT500 group (Figure 4). In contrast, the VT500 group had a conspicuous rise (*p* < 0.05) in the levels of 5-aminimidazole-4-carboxamide ribonucleotide (AICAR), guanosine monophosphate (GMP), guanosine, inosinic acid (IMP), uridine diphosphate glucose (UDP-glucose), and uridine diphosphate glucuronic acid (UDP-glucuronic acid); however the VT500 group decreased (*p* < 0.05) concentrations of hypoxanthine, fructose 6-phosphate (fructose 6P), and gluconic acid (Figure 4).

### 2.6. VT Affects the Expression of Genes Related to Hepatic Glucose Metabolism

**Figure 5:**
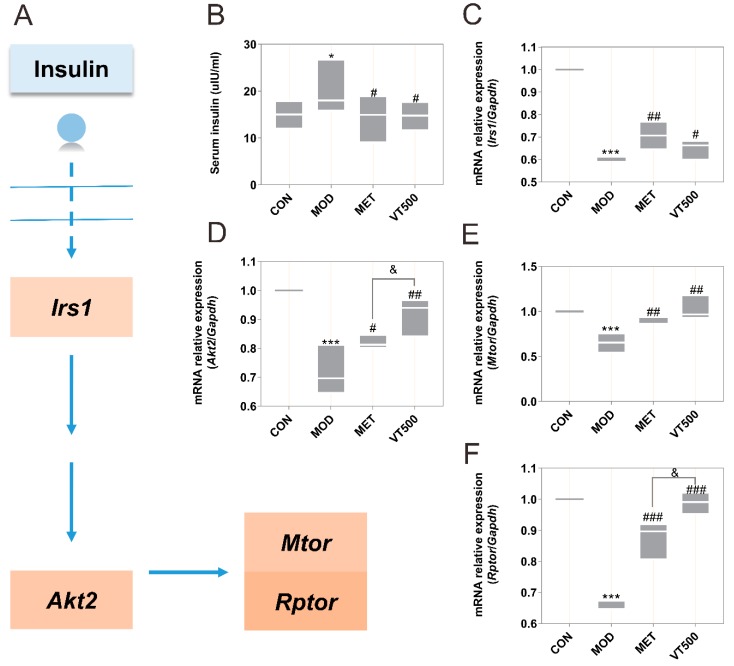
The effect of VT on the expression of genes involved in Akt signaling pathway. Insulin receptor substrate 1 (*Irs1*), RAC-beta serine/threonine-protein kinase (*Akt2*), mammalian target of rapamycin (*Mtor*), and regulatory-associated protein of Mtor (*Rptor*) were examined by quantitative real-time reverse transcription polymerase chain reaction (qRT-PCR), and the glyceraldehyde-3-phosphate dehydrogenase (*Gapdh*) gene, as a standard internal reference gene, was simultaneously examined for qRT-PCR normalization. Schematic diagram of the indicators related to the Akt signaling pathway in this study (**A**); the level of serum insulin in all groups (**B**); mRNA expression of *Irs1* (**C**), *Akt2* (**D**), *Mtor* (**E**) and *Rptor* (**F**) in the liver of each group. The data are presented as floating bars (min-max), and the CON group was used to standardize. * *p* < 0.05, *** *p* < 0.001 in comparison to the CON group, # *p* < 0.05, ## *p* < 0.01, ### *p* < 0.001 compared to the MOD group, & *p* < 0.05 contrasted to the MET group, *n* = 6, three replicates.

According to the biochemical indices and metabolomics results, the expression of some genes in the Akt signaling pathway was detected using quantitative real-time reverse transcription polymerase chain reaction (qRT-PCR) to further explore the effects of VT on glucose metabolism. A simplified diagram of the genes involved in the Akt signaling pathway is shown in Figure 5A. Although the elevated serum insulin in the MOD group may stimulate the insulin signaling pathway (Figure 5B), the results actually showed that the mRNA expression of insulin receptor substrate 1 (*Irs1*, Figure 5C), RAC-beta serine/threonine-protein kinase (*Akt2*, Figure 5D), mammalian target of rapamycin (*Mtor*, Figure 5E), and regulatory-associated protein of Mtor (*Rptor*, Figure 5F) was significantly decreased in the rats of the MOD group in comparison to those in the CON group rats (*p* < 0.05). As expected, after MET and VT treatments, all of these gene expressions have been significantly improved compared with T2D rats in the MOD group (*p* < 0.05); moreover, MET appeared to be more effective than VT in this regard. These findings suggest that VT improves glucose metabolism disorder via regulating the insulin/Irs/Akt/Mtor signaling pathway.

### 2.7. VT Regulates the Liver Expression Level of Proteins Involved in Glucose and Lipid Metabolism

**Figure 6:**
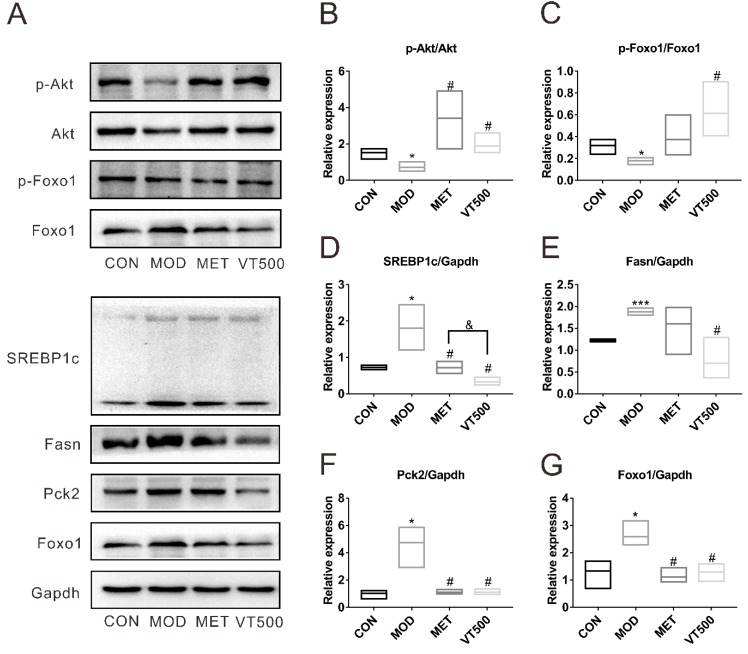
Effects of VT on the proteins involved in glucose and lipid metabolism. The expression of phosphorylated protein kinase B (p-Akt), Akt, phospho-Forkhead box protein O1 (p-Foxo1), Foxo1, sterol response element-binding protein 1c (SREBP1c), fatty acid synthase (Fasn), and phosphoenolpyruvate carboxykinase 2 (Pck2) was detected by WB, and Gapdh was simultaneously detected as a standard internal reference protein (**A**); the expression ratio of p-Akt to Akt in each group (**B**); the expression ratio of p-Foxo1 to Foxo1 in different group (**C**); the protein expression of SREBP1c in four groups (**D**); the protein expression of Fasn in each group (**E**); the protein expression of Pck2 in all groups (**F**); and the protein expression of Foxo1 in the respective groups (**G**). The data are presented as floating bars (min-max, three repetitions), and the CON group was used for standardization. * *p* < 0.05, *** *p* < 0.001 contrasted with the CON group, # *p* < 0.05 compared to the MOD group, and *p* < 0.05 compared with the MET group, *n* = 6.

The protein expression levels of phosphorylated protein kinase B (p-Akt), Akt, phospho-Forkhead box protein O1 (p-Foxo1), Foxo1, sterol response element-binding protein 1c (SREBP1c), fatty acid synthase (Fasn), phosphoenolpyruvate carboxykinase 2 (Pck2), and Gapdh were detected by Western blot (WB) (Figure 6A), and the protein expression ratio of the target protein to Gapdh was determined for the relative expression level of each protein. As shown in Figure 6, the expression ratios of p-Akt to Akt (Figure 6B) and p-Foxo1 to Foxo1 (Figure 6C) in the MOD group were markedly lower (*p* < 0.05) than that in the CON group, but both two ratios were significantly upregulated after VT treatment. Conversely, the absolute protein expression levels of SREBP1c (Figure 6D), Fsan (Figure 6E), Pck2 (Figure 6F), and Foxo1 (Figure 6G) in rats of the MOD group (*p* < 0.05) were substantially higher than that in the rats of CON group, while these proteins were significantly downregulated (*p* < 0.05) in the VT500 group compared with the MOD group. Therefore, the results indicated that VT may ameliorate glucose and lipid metabolism by regulating the Akt/Foxo1/Pck2 signaling pathway and downregulating the SREBP1c/Fasn signaling pathways.

## 3. Discussion

As a traditional functional tea, VT is widely used in the treatment of pharyngitis in southern China due to its remarkable anti-inflammatory activity. Additionally, VT is used as a health tea to protect the liver and to prevent hyperlipidemia and hyperglycemia. DHM is widely recognized as the main active constituent of VT [25,26,27,28,40] and is a quality control reference standard of VT (Figure S1). Previous research in our laboratory supported the traditional medicinal uses of VT and found that VT can effectively guard against insulin resistance and metabolic disorders caused by long-term intake of HFD [28,29,30]. The current study clarified the potential and mechanism of VT to ameliorate glucose and lipid metabolism disorder in T2D and provided a basis for the exploitation of VT as a hypoglycemic functional tea product.

In this study, the MOD group of rats developed hyperglycemia, dyslipidemia, hyperinsulinemia, insulin resistance and weight loss (Figure 1 and Figure 2), which agree with previous investigations [41,42], suggesting that the animal model of T2D with glucose and lipid metabolic syndrome was successfully established [43], and the occurrence of hyperinsulinemia may be due to resulting compensatory insulin release in the rats in the MOD group [42,44]. There was no difference in initial BW between the MOD group and the VT500 group; however, after four-weeks of VT intervention, weight gain and food utilization without hyperphagia of the VT500 group rats were higher than that in the rats in MOD group (Table 1 and Figure S2). There are also previous reports of weight gain after treatment [45]. The results suggested that VT may improve weight control in T2D rats by changing food utilization. What is different from our expectations is that the tea or water intake of rats in the MOD group and VT500 group was not significantly different. A possible reason for this result is that the demand for liquids in rats may be affected by the different contents and taste of tea and water, but the exact explanation needs further study (Figure S2) [46].

Furthermore, certain serum biochemical traits associated with liver function, glucose and lipid metabolism were significantly changed after VT treatment. The liver plays a vital role in the metabolism of various substances and is closely related to metabolic disorder in T2D. The serum ALT level and liver-to-BW ratio in the VT500 group were significantly lower than that in the MOD group (Figure 1 and Figure S2). These results consistently demonstrated the role of VT in improving liver function, which agreed with previous literature that indicated DHM could ameliorate nonalcoholic fatty liver disease [26]. Dyslipidemia is universally recognized to promote T2D development and cardiovascular complications in T2D [1,47]. The diagnostic criteria for dyslipidemia are considered high concentrations of serum TG, CHO, and LDL and low levels of serum HDL. Moreover, NEFA is used as a sensitive indicator for dyslipidemia; excessive concentrations of NEFA can heighten the prevalence of cardiovascular disease, metabolic syndrome and T2D [34,48,49]. The present results indicated that the VT500 group had lower serum levels of TG, CHO, LDL, and NEFA and higher levels of HDL than the MOD group, suggesting that VT has a good effect on improving dyslipidemia. These findings are also supported by the other research. Liu et al. conducted an 8-week animal experiment on HFD-fed LDL receptor deficient mice, and the results supported that DHM can significantly reduce serum CHO, TG, LDL levels, and increase serum HDL level [50]. Hyperglycemia is a major feature of diabetes. In addition to commonly used diagnostic criteria, including fasting blood glucose (FBG), insulin content, GTT and ITT, HbA1c levels have been recommended in recent years as an important criterion for diagnosing diabetes, based on a new understanding of the correlation between hyperglycemia and diabetes [51,52]. Additionally, the HOMA-IR index and QUICKI were performed to evaluate the development of T2D. Our results indicated that VT significantly reduced the levels of FBG, HbA1c and insulin, improved glucose tolerance, and accelerated insulin sensitivity (Figure 2). Similarly, the effect of DHM on improving insulin resistance and hyperglycemia has also been reported in other studies [28,53]. In this study, we found that VT reduced liver-to-BW ratio but increased the epididymal WAT-to-BW ratio and BW gain of T2D rats (Figure S2). A possible reason is that VT improved glycemic control, enhanced the action of insulin on fat, and regulated the storage and distribution of adipose tissue, which is supported by the results we mentioned above (Figure 1 and Figure 2, Figure S2) and another previous study [45]. The difference in epididymal WAT-to-BW ratio between the VT group and the CON group was not observed in our study, which suggests that VT only moderately increased epididymal WAT mass to combat excessive weight loss caused by STZ-induced T2D. Furthermore, the previous study has found that STZ-induced diabetes markedly decreased lipid content and LDL receptors amount in epididymal WAT, which can be corrected to normal after insulin treatment. However, STZ-induced diabetes or insulin treatment have no significant effect on hepatic LDL receptors [54]. And another study has showed that DHM significantly improved hyperlipidemia in LDL receptor deficient mice [50]. These studies indicate that VT may improve hyperlipidemia by regulating LDL receptors or LDL receptor-like function in epididymal WAT, resulting in a moderate increase in the epididymal WAT-to-BW ratio.

In addition to animal and cell experiments, some researchers have carried out clinical trials (CT) of VT to improve glucose and lipid metabolism, but the number of studies is very limited. After DHM treatment, Chen et al. [55] found that HOMA-IR index and the levels of serum glucose and LDL were significantly decreased, serum TG and CHO levels showed a downward trend in patients with nonalcoholic fatty liver disease. While the subjects did not develop diabetes, this study supports our findings to some extent. For patients with dyslipidemia, Liu et al. found that drinking VT can significantly reduce serum TG and CHO levels [56]. For T2D, a double-blind randomized CT was performed between VT and placebo tea, and subjects drank twice a day of VT or placebo tea for one month [57]. The results showed that VT significantly decreased FBG and glycated albumin levels in patients with T2D, which is consistent with our results. While no significant effects of VT on lipid levels were observed in this CT, serum CHO level was decreasing [57]. For the CT results different from our results, a possible reason is that rats with T2D and hyperlipidemia induced by HFD combined with STZ were used in our experiment, while subjects enrolled in this CT have normal Body Mass Index and no significant hyperlipidemia. This possibility is supported by Liu’s clinical research [56]. Another possible reason is that we replaced drinking water with VT; the full-time intervention resulted in long-lasting effects of VT for a relatively longer period of time. In summary, our results indicated that VT, as a functional food, has great potential as a supplementary treatment of T2D by improving insulin resistance and metabolic disorders, including glucose metabolism and lipid metabolism. Therefore, we believe that the mechanism of VT related to metabolic changes, especially the changes in glucose and lipid metabolism, and improvement of insulin resistance, are worthy of further investigation.

Metabolites have a variety of effects at the cellular level, thus, metabolites are considered to be effectors of molecular events that can help explain the molecular basis and physiological basis of diseases of interest [58]. While organismal metabolic processes are very complicated, we attempted to comprehensively understand the role of VT on metabolism. The liver is considered an important organ in the metabolic processes of the human body [59]. Therefore, we analyzed the changes in liver metabolites in rats with targeted metabolomics to expound the effect of VT on liver metabolites in T2D rats. This study focused on amino acid metabolism, purine metabolism, the glycolysis pathway, the pentose phosphate pathway, and the TCA cycle. The metabolites in the liver were identified and quantified by LC-MS, and 174 metabolites were detected. Then, a similarity analysis between the groups was performed by sPLS-DA (Figure 3) [60,61,62,63,64,65,66]. The comparison of the sPLS-DA score plot of the MOD and VT500 groups suggested that VT has a certain influence on liver metabolism, and the 29 metabolites with significantly altered concentration may reveal the mechanism of VT (Figure 4). KEGG pathway analysis was performed on the 29 key hepatic metabolites in the MOD and VT500 groups to clarify the actions of VT on metabolic pathways, and the results indicated that VT intervention mainly affected the amino acid metabolic pathway, the purine metabolic pathway, and glucose-related metabolic pathways in T2D rats (Table S1).

Several prospective studies have reported that amino acid metabolism and purine metabolism are closely related to diabetes and diabetic complications [67,68]. A previous study found that the glucose uptake of human forearm tissue decreased after threonine, leucine or isoleucine injection compared with control conditions, and the researchers concluded these amino acids may provide an alternative source of oxidized fuel [69]. Similarly, previous studies have also shown that the levels of tyrosine, phenylalanine [67,70], valine [71], glutamic acid [72], alanine, asparagine, methionine [73], aspartic acid [74], and proline [75] positively correlate with insulin resistance, glucose metabolic disorder and lipid metabolic disorder in T2D, while the levels of glycine and glutamine are inversely associated with the incidence of T2D [67,70,72]. Our data showed that VT reduced the levels of specific amino acids, including l-tyrosine, l-methionine, l-threonine, l-glutamic acid, l-phenylalanine and l-isoleucine, rather than increasing the concentrations of the amino acids that are inversely related to insulin resistance and T2D (Figure 4). In the purine synthesis pathway, the first nucleotide that is formed is IMP, which can be converted into adenosine monophosphate (AMP) or GMP, and guanosine is converted from GMP [76]. Insufficient levels of IMP, GMP and AMP will reduce the negative feedback regulation of purine synthesis and aggravate purine metabolic disorders, and these metabolites are very closely related to glucose and lipid metabolism [76,77]. We observed that the levels of IMP, GMP and guanosine, as well as AMP, in the rat liver increased significantly after VT intervention (Figure 4). Moreover, hypoxanthine is converted from IMP, and hypoxanthine is considered a key biomarker of purine metabolism of diabetes [78]. High concentrations of hypoxanthine are generally considered to exacerbate the development of diabetes [68,79,80]. Our data indicated that the concentration of hypoxanthine decreased significantly after VT supplement treatment. These findings suggested that VT can extensively regulate the liver metabolic function of T2D rats and reduce the risk of hepatic insulin resistance.

AICAR and AMP are important metabolites in purine metabolism and are also agonists of AMPK [81,82], which is a key issue in studies of T2D and related metabolic diseases [83]. Supplementation with AICAR can enhance glucose uptake and glycogen synthesis and improve insulin resistance and dyslipidemia [84,85]. In the present study, compared with the MOD group, AICAR significantly increased, and the level of AMP also had an increasing trend, after VT intervention (Figure 4). UDP-glucose is an activated form of glucose in nucleotide glucose metabolism and is used as a precursor for glycogen, sucrose lipopolysaccharide, and glycosphingolipids. UDP-glucuronic acid is converted from UDP-glucose and is also used in the synthesis of polysaccharides [59]. Our study found that VT can increase the contents of UDP-glucose and UDP-glucuronic acid (Figure 4). These results indicated that VT may improve the hyperglycemic symptoms of T2D by promoting glucose transport and the synthesis of glycogen and other carbohydrates. Studies have shown that certain glycolysis intermediates are increased in diabetic rats, such as fructose-6-phosphate and gluconic acid [86,87]. Additionally, the levels of fructose-6-phosphate and gluconic acid in the liver of the MOD group were increased, while VT reversed the change in metabolite concentrations, suggesting that VT can improve glucose metabolism disorder in T2D by regulating glycolysis disorders (Figure 4).

To understand more about the mechanisms of action of VT in improving insulin resistance, hyperglycemia and dyslipidemia, in addition to using targeted metabolomics to analyze changes in related metabolites, we also examined several important genes and proteins related to Akt signaling pathway, which is the main pathway involved in glucose and lipid metabolism (Figure 5 and Figure 6). We found that the expression levels of the *Irs1*, *Akt2*, *Mtor,* and *Rptor* genes in T2D rats were notably lower than those in CON group of rats. All of these genes were significantly upregulated after VT treatment, suggesting that VT downregulated serum insulin levels but increased insulin sensitivity and improved insulin resistance via the Irs/Akt/Mtor signaling pathway, which clarified the results of the ITT, QUICKI, and HOMA-IR at the genetic level. Akt protein is the key protein in the insulin signaling pathway and directly regulates glucose metabolism [88]. Foxo1 is a transcription factor that regulates glycogenolysis and gluconeogenesis through insulin signaling and controls adipogenesis [89]. Pck2 is a key protein regulating liver gluconeogenesis and a downstream protein of the Foxo1 signaling pathway. It is well known that insulin can inhibit the expression of Pck2 by activating the Akt/Foxo1 signaling pathway, which is one of the main ways for insulin to inhibit hepatic gluconeogenesis [90]. We discovered that VT promoted the phosphorylation of the Akt and Foxo1 proteins in the rat liver and reversed the T2D-mediated accumulation of Foxo1 protein in the nucleus. Then, we examined the level of Pck2 protein. As expected, the expression of Pck2 protein in the liver of VT group rats was significantly lower than that in the MOD group rats. In addition, studies have shown that NEFA can stimulate liver gluconeogenesis and increase the expression of PCK2 protein [91]. VT markedly decreased the level of NEFA in T2D rats, which may contribute to low level of Pck2 in the VT group. These findings combined with others in this study indicated that VT down-regulated Pck2 activity via the Akt/Foxo1 signaling pathway to inhibit liver gluconeogenesis. Based on the changes in the biochemical indicators, we found that VT can regulate lipid metabolism. As downstream proteins of the Akt/Foxo1 signaling pathway, the SREBP1c and Fasn proteins are major regulators of liver lipid homeostasis. Multiple studies have shown that overexpression of SREBP1c protein can increase level of TG, and is closely associated with increased liver fatty acid synthesis and hepatic steatosis; Fasn protein directly regulates fatty acid synthesis, and its expression is increased by stimulation of the overexpressed SREBP1c protein [24,92,93]. Moreover, SREBP1c and Fasn are highly expressed in the liver of a diabetic animal model [92,93], which is consistent with our results. On the contrary, VT significantly reduced the levels of both two proteins, suggesting that the process of liver fatty acid synthesis was inhibited by VT. This may also be a possible mechanism of a decrease in the liver-to-BW ratio in the VT group. Overall, VT improved insulin resistance, hyperglycemia and hyperlipidemia in T2D via the Akt signaling pathway.

However, there are some limitations in this research. First, although we used the same method to model T2D rats, differences in individual responses were inevitable, especially with small sample sizes. The sample size of each group should be expanded to better evaluate the effect of VT on T2D in the future. Second, due to the limitations of the experimental conditions, only one dose of VT was used to explore the potential of low-dose VT to improve hyperglycemia; different doses should be included in subsequent studies to provide a better basis for product development. Third, the targeted metabolomics used in this study was limited, and the influences of VT on metabolites related to lipid metabolism were not accurately demonstrated in this study, which is also a direction for follow-up research. Finally, the abovementioned effects of VT on hyperglycemia and hyperlipidemia were observed as a short-term intervention; however, for dietary supplements, the role of VT should be further evaluated with long-term intervention.

## 4. Materials and Methods

### 4.1. Vine Tea

The raw materials of VT were purchased from Guizhou Jiangkou Fanjingshan Yunfeng Wild Plant Development Co., Ltd. (Jiangkou, Guizhou, China). VT was prepared as follows: each 500 mg of crushed VT were immersed for 10 min in 1 L of boiling water; then, the hot VT solution was filtered to remove solid residues and cooled to room temperature. In addition, VT was freshly prepared every day and offered to the Wistar rats in the VT500 group on a free access basis.

### 4.2. Quality Control of VT

According to the method described above, VT was prepared, and the sample solution was obtained by filtration using a 0.22-μm Millipore membrane (Millipore Sigma, Burlington, MA, USA). DHM was precisely weighed in a 10-mL volumetric flask and dissolved in methanol (Fisher Scientific, Waltham, MA, USA) to a constant volume. After shaking, the standard solution (1 mg/mL) was prepared. The study performed quality control analysis of VT by Dionex UltiMate 3000 (Thermo Scientific, Waltham, MA, USA) equipped with a HSS-T3 column (Waters, Milford, MA, USA), and DHM (Shanghai Yuan Ye Biotechnology Co., Ltd., Shanghai, China) was used as a marker for standardization purposes. The experimental conditions were as follows: Column temperature of 30 °C; mobile phase of 0.1% formic acid-water (formic acid, Fisher Scientific, Waltham, MA, USA); detection wavelength of 278 nm; and flow rate controlled at 0.3 mL/min. The mobile phase conditions are listed in Table 2.

### 4.3. Experimental Animals

Forty male Wistar rats were purchased from SPF Biotechnology Co., Ltd. (approval No: SCXK (Jing)-2016-0002, Beijing, China) with an initial weight of approximately 200–220 g. All rats were raised under a light-controlled condition (12 h light/12 h dark) and in a temperature-controlled (23 ± 2 °C) room with food and water available in the animal center of The Institute of Medicinal Plant Development (approval No.: SYXK (Jing)-2013-0023, Beijing, China). Animal experiments were conducted in accordance with the guidelines established for the Care and Use of Laboratory Animals in the Beijing Government Guide and allowed by the Institutional Animal Use and Care Committee (IACUC) (approval No.: SLXD-20170301069).

### 4.4. Experimental Design

After adaptive feeding, we established the ND group (*n* = 10) and the HFD group (*n* = 30); all rats were randomly assigned to the two groups based on their BW. The ND group rats were fed the ND, while the HFD group rats were fed a HFD consisting of 45% fat (lard oil), 35% carbohydrate, and 20% protein for four-weeks (both feeds were purchased from Beijing HuaFukang Bioscience Co. Ltd., Beijing, China). Then, all rats were fasted for 12 h, and the rats in the HFD group received an intraperitoneal injection of small doses of STZ (30 mg/kg, Sigma, St. Louis, MO, USA; dissolved in citrate buffer, Solarbio, Beijing, China, pH 4.5), whereas the rats in the ND group received an equal volume of citrate buffer [43].

The ND rats and the HFD rats were refed a ND and a HFD respectively for 4 weeks to enhance the stability of T2D in the rats. Then, the blood glucose values of all rats were detected with the Roche blood glucose meter (Roche ACCU-CHEK, Basel, Switzerland) using blood collected from the tail vein after 8 h of fasting treatment. Rats in the HFD group with FBG higher than 7.8 mmol/L were considered to have successfully developed T2D. Then, these T2D rats were randomly grouped into the following 3 treatment groups according to FBG level: (1) The MOD group (allowed to freely drink distilled water + HFD diet, *n* = 6); (2) the MET group (allowed to freely drink distilled water + HFD diet + metformin (200 mg/kg/d metformin, Sino American Shanghai Squibb Pharmaceutical Ltd., Shanghai, China), *n* = 6); and (3) the VT500 group (allowed to freely drink 500 mg/L VT + HFD diet, *n* = 6). Four rats were randomly removed from the CON group to maintain *n* = 6, and the CON group rats consumed a ND and were allowed to freely drink distilled water.

After 4 weeks of treatment, all rats were sacrificed; 10% chloral hydrate (3 mL/kg i.p., Sigma, St. Louis, MO, USA) was used to reduce pain. The following samples were collected from the rats: abdominal aorta blood, liver tissue and epididymal WAT. The blood specimens were allowed to clot for at least 2 h; then, the serum was separated and collected under conditions of low speed centrifugation and low temperature (3000 rpm/min, 4 °C, 10 min, SIGMA 3K15, Osterode am Harz, Germany) and frozen at −80 °C (Thermo Scientific Forma 900 series, Waltham, MA, USA) until biochemical analysis. The weights of the liver and epididymal WAT were promptly measured after they were removed from the rats, and the samples immediately frozen in liquid nitrogen before storage for further experiments. Moreover, the ratios of the liver-to-BW and the epididymal WAT-to-BW of all groups were calculated to determine the effect of VT on the liver and epididymal WAT of rats. In addition, FI (g/day/rat) and water intake (ml/day/rat) were recorded daily. The BW of the animals were recorded every week.

### 4.5. GTT and ITT

GTT was performed on all animals using Roche ACCU-CHEK (Basel, Switzerland) during the last week of the experiment after 8 h of fasting. Blood specimens were obtained from the tail vein at 5 time points (0, 30, 60, 90, and 120 min) after 50% glucose solution injection (i.p., 2 g/kg BW, d-(+)-glucose, Sigma, St. Louis, MO, USA) [94]. ITT was carried out after 4 h of fasting, and the method and time points of blood sample collection was consistent with those for GTT, except that all rats were injected with insulin (s.c., 0.75 IU/kg BW, Eli Lilly and Company, Indianapolis, IN, USA) [95].

### 4.6. Serum Biochemical Experiments

The levels of GLU, HbA1c, TG, HDL, LDL, CHO, ALT (assay kits obtained from from Biosino Bio-Technology and Science Inc., Beijing, China), and NEFA (Beijing Strong Biotechnologies, Inc., Beijing, China) in the serum were measured with an AU480 Automatic Biochemical Analysis System (Beckman Coulter, Miami, FL, USA) using assay kits. Additionally, insulin was detected by Beijing Sino-UK Institute Biological Technology (Beijing, China). The HOMA-IR index was calculated as follows:

HOMA-IR = [fasting glucose (mmol/L) × fasting insulin (mIU/L)]/22.5,
(1)

The QUICKI was determined by the following formula [96]:

QUICKI = 1/[log fasting insulin (mIU/L) + log fasting glucose (mg/dL)],
(2)

### 4.7. Hepatic Metabolite Profiling

Based on a previous report, we prepared liver extracts and used pulse-acquire sequences to identify liver metabolites. [39]. A Q Exactive Orbitrap (Thermo Scientific, Waltham, MA, USA), loaded with an amide column (Waters, Milford, MA, USA), was used in the sample analysis, and the parameter settings were also based on previous reports [97]. An in-house MS/MS database containing more than 700 compounds was used to identify metabolites.

TSQ Quantiva (Thermo Scientific, Waltham, MA, USA; matching C18 column, Waters, Milford, MA, USA) was used for comprehensive metabolomics analysis. A solution of 100% methanol was used as the mobile phase and was increased from 5% to 90% over 25 min. The stationary phase consisted of 10 mM tributylamine and 15 mM acetic acid in H_2_O. The other parameters were all set according to previous reports [98]. The data acquisition included all of the positive and negative ion mode data. Thermo Xcalibur software (Thermo Scientific, Waltham, MA, USA) was used to analyze the raw data obtained from liver extracts; this program can transform spectral data into standardized peak intensities, multiple pairs of retention times and peak areas aligned with the same mass. Then, the results were Pareto scaled and mean centered. This analysis mainly focused on amino acids, purine metabolism, glycolysis, the pentose phosphate pathway, and the TCA cycle. The processed data were analyzed by MetaboAnalyst 4.0 (http://www.metaboanalyst.ca) for sPLS-DA.

### 4.8. QRT-PCR

The RNA from the liver tissues of rats (*n* = 6) was prepared by the RNAiso Plus Kit (Takara, Tokyo, Japan). We measured the concentration of mRNA in different groups using a NanoDrop 2000 (Thermo Scientific, Waltham, MA, USA) and performed reverse transcription after balancing the concentrations of total mRNA in each group using the PrimeScript^TM^ RT Reagent Kit (Perfect Real Time) (Takara, Tokyo, Japan). Then, real-time PCR was carried out according to the instructions of SYBR^®^ *Premix Ex Taq*^TM^ II (Tli RNaseH Plus) (Takara, Tokyo, Japan) on a detection system (BioRad CFX96™ Real-Time System, Hercules, CA, USA). Primer sequences are shown in Table 3.

### 4.9. WB Analysis

WB analysis was performed according to previous reports [97]. The partial liver tissues were homogenized with a protein extraction reagent, and quantitative denatured proteins were separated by 6–10% SDS-PAGE (RIPA Lysis Buffer, Micro BCA Protein Assay Kit and Tricine-SDS-PAGE Gel Kit, Beijing CoWin Biotech Co. Ltd., Beijing, China) and transferred to a PVDF membrane (Immobilon^®^ PVDF Membranes, Millipore Sigma, Burlington, MA, USA) for 100–150 min at 200 mA. The membranes were blocked with BSA blocking buffer (Beyotime Biotechnology Co. Ltd., Beijing, China) for 1 h at room temperature and incubated overnight at 4 °C with primary antibodies [p-Akt (ab192623), Akt (ab179463), SREBP1c (ab3259), Fasn (ab128870), Pck2 (ab70359), Gapdh (ab8245), Abcam, Cambridge, MA, USA; p-Foxo1 (#9461); Foxo1 (#2880), Cell Signaling Technology, Danvers, MA, USA]. Then, the membrane was washed with TBST (3 washes for 10 min each) and incubated with the appropriate secondary antibodies (Beijing CoWin Biotech Co. Ltd., Beijing, China) for 1 h at room temperature. After washing with TBST (5 washes for 5 min each), the protein bands were visualized and semi-quantified using a BIO-RAD ChemiDoc^TM^ MP Imaging System (Bio-Rad, Hercules, CA, USA) with a supporting system (ImageLab., Bio-Rad, Hercules, CA, USA).

### 4.10. Statistical Analysis

The results are presented as the mean ± standard deviation (SD). An unpaired *t*-test and one-way ANOVA were used to determine significant differences between two groups and multiple groups, respectively. *p* < 0.05 was judged as significant. GraphPad Prism 7 software (GraphPad Software, San Diego, CA, USA) was used for constructing figures, and MetaboAnalyst 4.0 (http://www.metaboanalyst.ca) was used for analyzing metabolomics data.

## 5. Conclusion

This study determined that VT has great potential as a traditional tea beverage in the supplementary treatment of T2D and also provided a basis for the potential commercial application of VT. A HFD combined with low-dose STZ successfully induced a rat model of T2D, while VT significantly improved insulin resistance and metabolic disorder in T2D rats, mainly including glucose metabolism and lipid metabolism. Metabolomics, qRT-PCR and WB were used to further explore the possible mechanisms of the effects of VT as a supplementary treatment for metabolic disorder in T2D.The metabolomics results suggested that VT ameliorated T2D by extensively improving liver metabolic disorders, promoting glycogen synthesis and glycolysis. Furthermore, qRT-PCR results indicated that VT influenced the expression of *Irs1*, *Akt2*, *Mtor* genes in the Akt signaling pathway, suggesting that VT enhanced insulin signaling and improved liver insulin resistance. The WB results suggested that VT improved insulin resistance, inhibited gluconeogenesis through the Akt/Foxo1/Pck2 signaling pathway, and reduced fatty acid synthesis via the SREBP1c/Fasn signaling pathways.

## Figures and Tables

**Table 1:** Effect of VT on body weight (BW), food intake (FI), and food utilization rate in rats with T2D.

Group	Initial BW (g)	Initial BW before Drug (VT) Treatment (g)	Final BW (g)	BW Gain (g)	FI Per Day (g)	Food Utilization Rate (%)
CON	250.1 ± 15.2	549.5 ± 92.1	607.7 ± 75.4	58.2 ± 23.4	25.9 ± 3.4	7.2 ± 2.9
MOD	247.2 ± 15.1	429.8 ± 63.1 *	437.0 ± 52.0 **	7.2 ± 17.3 **	26.6 ± 7.0	0.9 ± 2.1 **
MET	247.2 ± 15.1	451.0 ± 49.1	491.2 ± 53.8	40.2 ± 26.3 #	25.1 ± 6.4	5.2 ± 3.4 #
VT500	247.2 ± 15.1	443.8 ± 52.5	478.5 ± 73.5	34.7 ± 24.2 #	25.5 ± 5.0	4.4 ± 3.1 #

Note: The data are presented as the mean ± standard deviation (SD). * *p* < 0.05, ** *p* < 0.01 compared to the CON group; # *p* < 0.05 compared to the MOD group, *n* = 6.

**Table 2:** The mobile phase conditions of ultra-high-performance liquid chromatography (UPLC).

Time (min)	0.1% Formic Acid Solution in Water (%)	Methanol (%)
0	75	25
17	40	60
19	0	100
22.2	75	25

**Table 3:** Primer sequences used for the analysis of gene expression with real-time PCR.

Gene	Primers	5′ Primer Sequence 3′
*Irs1*	Forward	CAT GAG CCC CAA GAG TGT ATC
	Reverse	CCA ATG TCA GGA GAG CAA CTA C
*Akt2*	Forward	CCG AGT CCT ACA GAA TAC CAG
	Reverse	ACT CCA TCA CAA AGC ACA GG
*Mtor*	Forward	ATC CTT AAT CTG TTG CCC CG
	Reverse	ATG GTG TCT TGC AGG TAC TG
*Rptor*	Forward	TGG TGC CTG GAG TCA CAC TG
	Reverse	GCA CAT TCC AGG CGA TGG TG
*Gapdh*	Forward	GAC ATG CCG CCT GGA GAA AC
	Reverse	AGC CCA GGA TGC CCT TTA GT

## Supplementary Materials

The following are available online, Figure S1: determination of DHM content in VT by UPLC, Figure S2: effect of VT on the BW, FI, water (tea) intake, liver-to-BW ratio and epididymal white adipose tissue (WAT)-to-BW ratio of each group (DOCX), Table S1: pathway impact analyses of key hepatic metabolites (*p* < 0.05) between the VT500 group and MOD group by MetaboAnalyst 4.0 (DOCX).
