# Supplementary material for: Dietary Supplementation of Vine Tea Ameliorates Glucose and Lipid Metabolic Disorder via Akt Signaling Pathway in Diabetic Rats
Source: Molecules. 2019 May 15;24(10):1866. doi: 10.3390/molecules24101866 (PMC6571802; doi:10.3390/molecules24101866)
Supplement: Supplementary file 1 [file molecules-24-01866-s001.pdf]

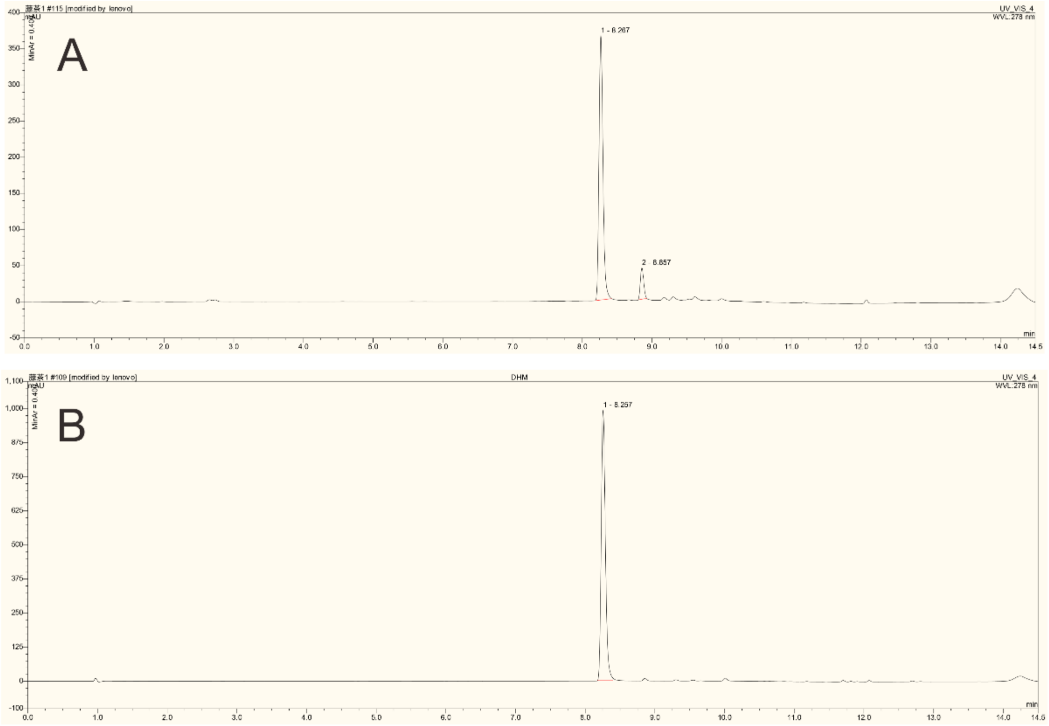

Figure S1. Determination of dihydromyricetin (**DHM**) content in VT by UPLC. Chromatogram of DHM in VT (**A**). Chromatogram of DHM (**B**).

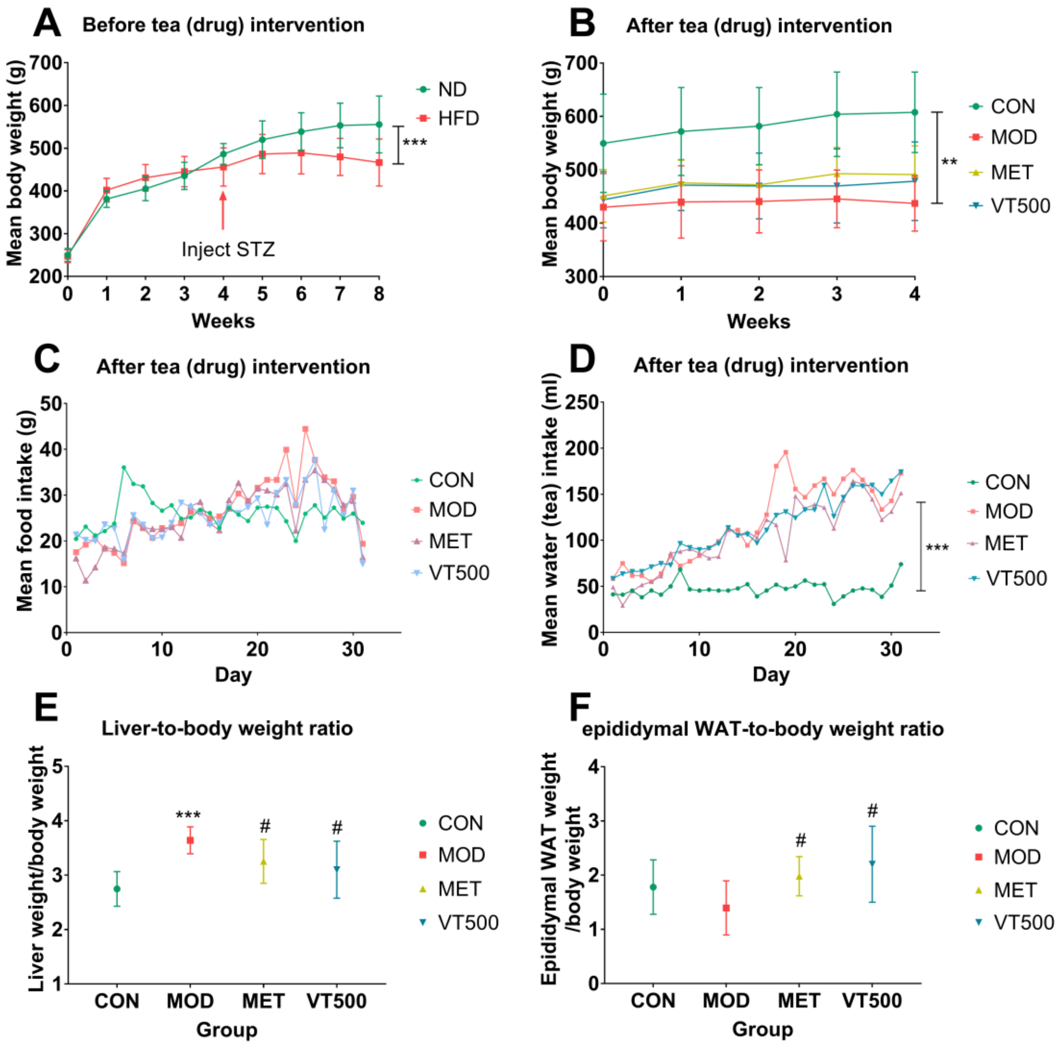

Figure S2. Effect of VT on the BW, FI, water (**tea**) intake, liver-to-BW ratio and epididymal white adipose tissue (**WAT**)-to-BW ratio of each group. Changes in BW during the 8-week modeling process (**ND**, **n = 10**; **HFD**, **n = 30**, **A**); changes in BW during the 4 weeks of treatment, n=6 (**B**); changes in food intake during the 4 weeks of treatment, n = 6 (**C**); changes in water (tea) intake during the 4 weeks of treatment, n=6, (**D**); liver-to-BW ratio of each group, n = 6 (**E**); epididymal WAT-to-BW ratio of each group, n = 6 (**F**). Data are presented as the mean ± SD. ** $P < 0.01$, *** $P < 0.001$ compared to the CON group; # $P < 0.05$ compared to the MOD group.

Table S1. Pathway impact analyses of key hepatic metabolites (**p < 0.05**) between the VT500 group and MOD group by MetaboAnalyst 4.0.

NO.	Pathway name	Total Compound	Hits	Raw p	FDR	Impact
1	D-Glutamine and D-glutamate metabolism	5	1	0.001247	0.004208	1.00
2	Alanine, aspartate and glutamate metabolism	24	1	0.001247	0.004208	0.26
3	Arginine and proline metabolism	44	1	0.001247	0.004208	0.09
4	Glutathione metabolism	26	1	0.001247	0.004208	0.06
5	Histidine metabolism	15	1	0.001247	0.004208	0.00
6	Butanoate metabolism	20	1	0.001247	0.004208	0.00
7	Porphyrin and chlorophyll metabolism	27	1	0.001247	0.004208	0.00
8	Nitrogen metabolism	9	1	0.001247	0.004208	0.00
9	Sphingolipid metabolism	21	1	0.002110	0.005698	0.02
10	Glycerophospholipid metabolism	30	1	0.002110	0.005698	0.00
11	Riboflavin metabolism	11	2	0.002734	0.006710	0.33
12	Amino sugar and nucleotide sugar metabolism	37	5	0.004709	0.010594	0.13
13	Galactose metabolism	26	2	0.005826	0.012100	0.00
14	Aminoacyl-tRNA biosynthesis	67	4	0.008002	0.015432	0.00
15	Purine metabolism	68	6	0.009059	0.016306	0.30
16	Nicotinate and nicotinamide metabolism	13	3	0.011847	0.019992	0.17
17	Cysteine and methionine metabolism	28	1	0.014437	0.022929	0.09
18	Pantothenate and CoA biosynthesis	15	1	0.016330	0.024495	0.02
19	Glycine, serine and threonine metabolism	32	1	0.020004	0.027522	0.00
20	Phenylalanine, tyrosine and tryptophan biosynthesis	4	1	0.023445	0.027522	0.50
21	Tyrosine metabolism	42	1	0.023445	0.027522	0.14
22	Ubiquinone and other terpenoid-quinone biosynthesis	3	1	0.023445	0.027522	0.00
23	Phenylalanine metabolism	9	1	0.023445	0.027522	0.00
24	Pyrimidine metabolism	41	1	0.038229	0.043008	0.07

25	Pentose and glucuronate interconversions	14	2	0.044200	0.044200	0.64
26	Ascorbate and aldarate metabolism	9	2	0.044200	0.044200	0.40
27	Starch and sucrose metabolism	23	2	0.044200	0.044200	0.24
